# T-cell infiltration and its regulatory mechanisms in cancers: insights at single-cell resolution

**DOI:** 10.1186/s13046-024-02960-w

**Published:** 2024-02-02

**Authors:** Wenhui Yang, Shimao Liu, Mengyun Mao, Yandong Gong, Xiaohui Li, Tianyu Lei, Chao Liu, Shikai Wu, Qinyong Hu

**Affiliations:** 1 Department of Oncology, Renmin Hospital of Wuhan University, Wuhan, 430060 China; 2 State Key Laboratory of Experimental Hematology, Senior Department of Hematology, Fifth Medical Center of Chinese PLA General Hospital, Beijing, 100071 China; 3 Department of Medical Oncology, Peking University First Hospital, Beijing, 100034 China; 4 Department of Radiation Oncology, Peking University First Hospital, Beijing, 100034 China

**Keywords:** Single-cell RNA sequencing, Tumor microenvironment, T-cell infiltration, “hot tumors” and “cold tumors”, Stromal cells

## Abstract

Tumor-infiltrating T cells recognize, attack, and clear tumor cells, playing a central role in antitumor immune response. However, certain immune cells can impair this response and help tumor immune escape. Therefore, exploring the factors that influence T-cell infiltration is crucial to understand tumor immunity and improve therapeutic effect of cancer immunotherapy. The use of single-cell RNA sequencing (scRNA-seq) allows the high-resolution analysis of the precise composition of immune cells with different phenotypes and other microenvironmental factors, including non-immune stromal cells and the related molecules in the tumor microenvironment of various cancer types. In this review, we summarized the research progress on T-cell infiltration and the crosstalk of other stromal cells and cytokines during T-cell infiltration using scRNA-seq to provide insights into the mechanisms regulating T-cell infiltration and contribute new perspectives on tumor immunotherapy.

## Introduction

The tumor microenvironment (TME) is a key component of the multistage, and multipathway abnormal growth process known as tumorigenesis [1–4]. The TME includes multiple immune cell types, cancer-associated fibroblasts (CAFs), endothelial cells, and other tissue-resident cell types [5–7]. T cells, including CD4^+^ and CD8^+^ T cells, are an important component of the TME [8]. In addition, they are crucial to the response of the immune system to immunotherapy [9, 10].

Tumors can be classified as “hot” or “cold” tumors based on the density of lymphocyte infiltration in the TME [11–13]. “Hot tumors” are characterized by high lymphocyte infiltration in the TME, whereas “cold tumors” have few infiltrating lymphocytes and are immune ignorant. In addition, “immune excluded” and “immune desert” are the two other types of immune altered tumors [14]. The “immune excluded” type has a large number of lymphocytes at its edge which cannot infiltrate into the tumor, whereas the “immune desert” type has a very low density of lymphocytes at the center and periphery [9]. Immunotherapy has become a cutting-edge method of treating tumors in recent years. The clinical efficacy of immunotherapy is closely linked to the baseline antitumor immune response and the activation of immune responses within tumors. The immunologic status is substantially different between “hot " and “cold” tumors, leading to variations in the response to immunotherapy [15]. “Hot tumors” are more clinically responsive to immunotherapy. Therefore, the transformation of “cold” into “hot” tumors has emerged as a prominent focus in the field of immunotherapy [9].

Bulk sequencing analysis has provided voluminous data for exploring the factors influencing T-cell infiltration within tumors. However, some distinctive cell subtypes or states may go unnoticed because these data are based on specific cell populations [16], For instance, certain tumor-associated macrophages (TAMs) [17], dendritic cells (DCs) [18], and possible cancer stem cells, important during T-cell infiltration into a tumor, may not have been detected during bulk sequencing. Single-cell RNA sequencing (scRNA-seq) has gained enormous popularity in recent years. ScRNA-seq is a high-resolution method for studying single cells that can provide insight into their genetic and molecular makeup and the degree of cell heterogeneity [19, 20]. The technique allows us to reveal the states and functions of individual cells, and identify unique cell subtypes or states in tumors [21–24]. Thus, we can analyze the factors that influence “hot” and “cold” tumors [25]. It is critical to decide the choice of immunotherapy approaches, immune monitoring, and survival prognosis prediction. Moreover, an understanding of the transition between “hot” and “cold” tumors may potentially advance the immunotherapeutic approaches for a wide range of patients, thereby enhancing their survival. Here, we reviewed studies using scRNA-seq methods to analyze the TME in “hot” and “cold” tumors. We focused on the patterns of T-cell infiltration in the TME and the mechanisms by which other stromal cells and cytokines influence T-cell infiltration.

## Intertumoral heterogeneity of infiltrating T cells

“Hot tumors” are characterized by high T-cell infiltration with a large number of T cells in the tumor tissues and a strong antitumor immune response. For example, highly vascularized clear cell renal cell carcinoma (ccRCC) has a high level of infiltrating immune cells, mainly CD8^+^ T cells [20]. Similarly, non-small cell lung cancer (NSCLC) is characterized by a significant increase in T-cell infiltration, which indicates a potent immune response inside the TME. ScRNA-seq of T-cell profiles in NSCLC revealed a high proportion of pre-exhausted and exhausted CD8^+^ T cell subsets and numerous highly migratory effector T cells in tumor tissues, which correlated with good prognoses. However, this phenomenon was not observed in the scRNA-seq of T-cell profiles in squamous cell lung carcinoma [26]. Melanoma, another “hot tumor,” is infiltrated by CD4^+^ and CD8^+^ T cells, which have a crucial role in antitumor therapy. However, the presence of regulatory T cells (Tregs) within the tumor hinders the ability of the immune system to effectively combat the tumor [27, 28]. Breast cancer (BC) was earlier not considered immunologically active. However, comprehensive research on the BC microenvironment revealed a significant infiltration of T cells despite the low number of T cells in the TME [29]. Breast tumor lesions showed a high concentration of immune cells, mainly CD3^+^ T cells, indicating a robust immune response [30]. However, the levels of CD4^+^CD25^+^FOXP3^+^ and follicular Tregs were significantly increased in both the peripheral blood and breast tissues of patients with BC in all cancer subtypes [31]. This increase may contribute to tumor progression and metastasis.

Compared with these high T-cell infiltrating tumors, ovarian, prostate, pancreatic, colorectal, and gastric cancers have limited T-cell infiltration. Therefore, these tumors have immunosuppressive TME and are less responsive to immunotherapy. The density of T-cell infiltration in prostate cancer tissues is reduced compared with that in benign prostate hyperplasia, suggesting that prostate cancer progression is accompanied by a significant suppression of the immune system [32]. Ovarian cancer tissues have few infiltrating T cells, and TAMs and Tregs are the major infiltrating subpopulations mediating the immunosuppressive microenvironment [33]. ScRNA-seq analysis indicates T and NK cell dysfunction, low cytotoxic T cell (CTL) infiltration, and a predominance of immunosuppressive myeloid cells and macrophages in pancreatic tumors, indicating an immunosuppressive TME [34]. A study on T cells from various solid tumors revealed significantly lower T-cell infiltration in colorectal and gastric adenocarcinomas compared with that in renal clear cell, thyroid, and lung adenocarcinomas [35]. The low PD-L1 expression and decrease in tumor-infiltrating lymphocyte recruitment were consistent with an immunosuppressive TME. Therefore, T cells infiltrate tumors as the most predominant immune component, and immune heterogeneity is ubiquitous in almost all solid tumors and closely associated with the progression of tumors and the response to antitumor therapy.

## Intratumoral heterogeneity of infiltrating T cells and corresponding immunophenotypes

The heterogeneity of tumor-infiltrating T cells exists not only in different tumors but also in the same type of tumors. T-cell infiltration is different among various phenotypes of the same tumor. BC can be classified into four subtypes based on molecular staging: Luminal A, Luminal B, HER2^+^, and triple-negative breast cancer (TNBC) subtypes. Compared with the luminal A and B subtypes, the HER2^+^ BC and TNBC subtypes show abundant T-cell infiltration, and activation of T-cell-mediated immune responses enhances immune-related antitumor activity in these subtypes [36]. Some authors have classified BC into three immune subtypes, namely BC-ImH, BC-ImL, and BC-ImM based on the clustering analysis of bulk-seq tumor and scRNA-seq datasets with different immune signature scores. The TNBC and HER2^+^ BC subtypes with high T-cell infiltration have strong immune responses and better clinical outcomes, corresponding to BC-ImH with high immune scores. The HR^+^BC subtype with low T-cell infiltration has a weak immune response and worse clinical outcomes, corresponding to BC-ImL with low immune scores [37]. In addition, the TNBC phenotype is subdivided into three clusters: the “immune-desert” cluster with the lowest level of T-cell infiltration (Cluster 1), the “innate immune-inactivated” cluster with resting innate immune cell and non-immune stromal cell infiltration (Cluster 2), and the “immune-inflamed” cluster with the highest degree of T-cell infiltration (Cluster 3) [38]. Cluster 2 lacking T-cell infiltration and Cluster 1 having low T-cell infiltration may be associated with an underlying immune escape mechanism. Similarly, Wang et al. [39] classified patients with hepatocellular carcinoma from The Cancer Genome Atlas (TCGA) dataset into three immune subtypes, S1, S2, and S3. The S1 subtype was characterized as a “hot tumor” type with high T-cell infiltration, the highest levels of activated T-cell markers, and the best prognostic outcome. The S2 subtype was described as a “cold tumor” type with the lowest level of immune cell infiltration, and the S3 subtype was characterized as an “immunosuppressive tumor” type with high immune cell infiltration but predominant expression of the immunosuppressive genes with the worst prognosis. The authors characterized the tumor immune infiltration status among different phenotypes of patients with hepatocellular carcinoma by integrating scRNA-seq and multi-omics datasets and revealed the molecular heterogeneity of T-cell infiltration within tumors.

The immune infiltration status of the same tumor (such as the primary and metastatic lesions of a tumor) also varies in an individual, and this spatial heterogeneity can be manifested in different sites or organs. The TME is distinctly heterogeneous at the different primary sites in patients with melanoma. Compared with cutaneous melanoma samples, acral melanoma samples showed a markedly immunosuppressed state with a decrease in cytotoxic CD8^+^ T-cell infiltration and an increase in the infiltration of Tregs and exhausted CD8^+^ T cells [40]. Melanoma can also be divided into melanoma brain metastases (MBM) and leptomeningeal melanoma metastases (LMM) based on the site of tumor metastasis. LMM tissues had a high proportion of CD4^+^ T cells (especially exhausted and apoptotic CD4^+^ T cells) and low infiltration of CD8^+^ T cells. In contrast, MBM samples had a low proportion of CD4^+^ T cells but a high proportion of CD8^+^ T cells [41]. Therefore, the type and extent of T-cell infiltration within the tumor are spatially heterogeneous, influencing tumor progression and treatment response. In addition, analysis of the immune microenvironment of primary ovarian cancer and omental metastatic samples revealed differences in T-cell infiltration, recognition, and expansion. Quantitative and qualitative “cold” patterns are the two “immune cold” patterns in ovarian cancer. T cells infiltrating the primary ovarian foci have higher tumor specificity but are in an exhausted state and low numbers accompanied by immunosuppressive Treg infiltration in a quantitative “cold” pattern. In contrast, numerous T cells infiltrating the omental metastases are mostly in a naïve and immune memory state and act as bystander T cells with non-tumor specificity in qualitative “cold” patterns [42]. Compared with high-grade epithelial ovarian cancer, low-grade cancer has higher intraepithelial CD8^+^ and CD4^+^ T-cell infiltration and CD8^+^/CD4^+^ ratio [33]. Olalekan et al. [43] stratified ovarian cancer samples according to the degree of T-cell infiltration in scRNA-seq studies and identified TOX-expressing resident memory CD8^+^ T cell clusters and granulysin-expressing CD4^+^ T cell clusters in the high T-cell infiltration group. T-cell infiltration patterns in ovarian cancer were further classified into immune-infiltrated, excluded, and desert patterns. Immune-infiltrated tumors have more activated CD4^+^ T cells and Tregs. Resting *IL-7R* CD4^+^ T cells are significantly enriched in immune-excluded tumors, whereas immune-desert tumors lack T cells and are mainly enriched in monocytes and immature macrophages [44]. High T-cell infiltration is associated with better clinical outcomes and prognoses. Therefore, we should not only study the T-cell infiltration status of different tumors but also analyze the intratumoral heterogeneity of T-cell infiltration and corresponding phenotype to provide a reference for deciding optimal therapeutic strategies.

## Co-infiltration of T cells with other cells implies cellular crosstalk within the tumor

**Fig. 1:**
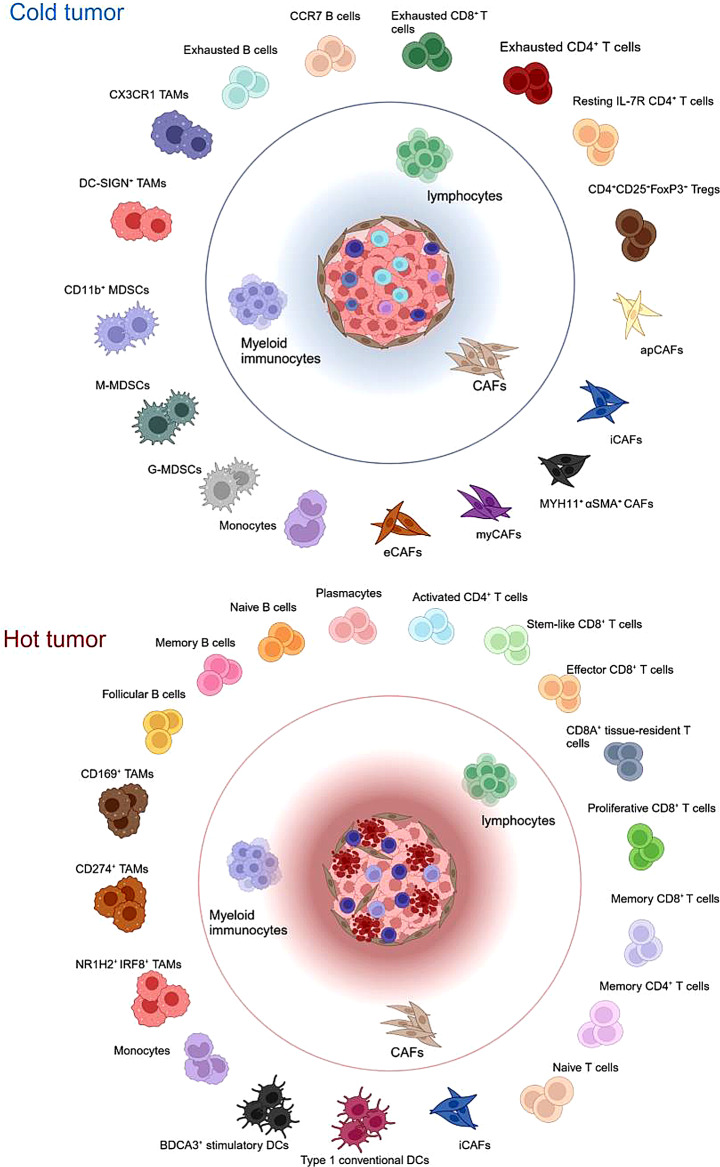
Summary of cells associated with T-cell infiltration in the “cold tumors” and “hot tumors” microenvironment by single-cell sequencing. (Figure was created with BioRender.com). apCAFs, Antigen-presenting cancer-associated fibroblasts; DCs, dendritic cells; eCAFs, Extracellular stromal cancer-associated fibroblasts; G-MDSCs, Granulocyte-like myeloid-derived suppressor cells; iCAFs, Inflammatory cancer-associated fibroblasts; myCAFs, Cancer-associated myofibroblasts; MDSCs, Myeloid-derived suppressor cells; M-MDSCs, Monocytic myeloid-derived suppressor cells; Tregs, Regulatory T cells; TAMs, Tumor-associated macrophages

The intertumor and intratumor heterogeneity of T-cell infiltration can be attributed to the tumor and its complex TME. ScRNA-seq allows researchers to precisely resolve the crosstalk between various cells within a tumor [45–48]. Therefore, the different mechanisms of T-cell infiltration can be analyzed by comparing the differences in cellular composition between “cold” and “hot” tumors (Fig. 1).

The intertumor heterogeneity of T-cell infiltration reveals variable crosstalk mechanisms between different tumors and T cells [49]. For example, tumor cells can reduce their immunogenicity by downregulating the expression of MHC molecules, thereby inhibiting T-cell recruitment [50, 51]. CAFs, a major type of stromal cells in the TME, can regulate T-cell infiltration and distribution through complex mechanisms. Grout et al. [52] found that NSCLC tumor foci infiltrated by a low number of CD8^+^ T cells were enriched in *MYH11*^+^*αSMA*^+^ CAFs. The authors also discovered a potential association between the *MYH11*^+^*αSMA*^+^ CAF subpopulation and the evasion of T cell-mediated immune responses within the TME. In addition, immune profiling of different tumor types reveals that the TMEs of “hot tumors” are predominantly enriched in effector B cells, NK cells, and M1 macrophages. Conversely, “cold tumors” are predominantly enriched in Tregs, M2 macrophages, and myeloid-derived suppressor cells (MDSCs) [14]. Hornburg et al. [44] reported that immune-desert tumors with minimal T-cell infiltration are characterized by an abundance of MDSC-like cells. In contrast, “hot tumors” with high T-cell infiltration are enriched in plasma cells, B cells, *NR1H2*^+^*IRF8*^+^ macrophages, and *CD274*^+^ macrophages. Taken together, “cold” and “hot” tumors are enriched with different cell subpopulations. The use of scRNA-seq technology for investigating the intercommunication among various cell subpopulations and T cells within tumors can help identify the underlying mechanisms of the development of “cold tumors.” This information can facilitate the conversion of “cold” into “hot” tumors for therapeutic purposes, enabling the use of immunotherapy to treat a large number of patients [53, 54].

## Regulatory mechanisms of T-cell infiltration at single-cell resolution

### Tumor cells evade killing by T cells through multiple mechanisms

**Fig. 2:**
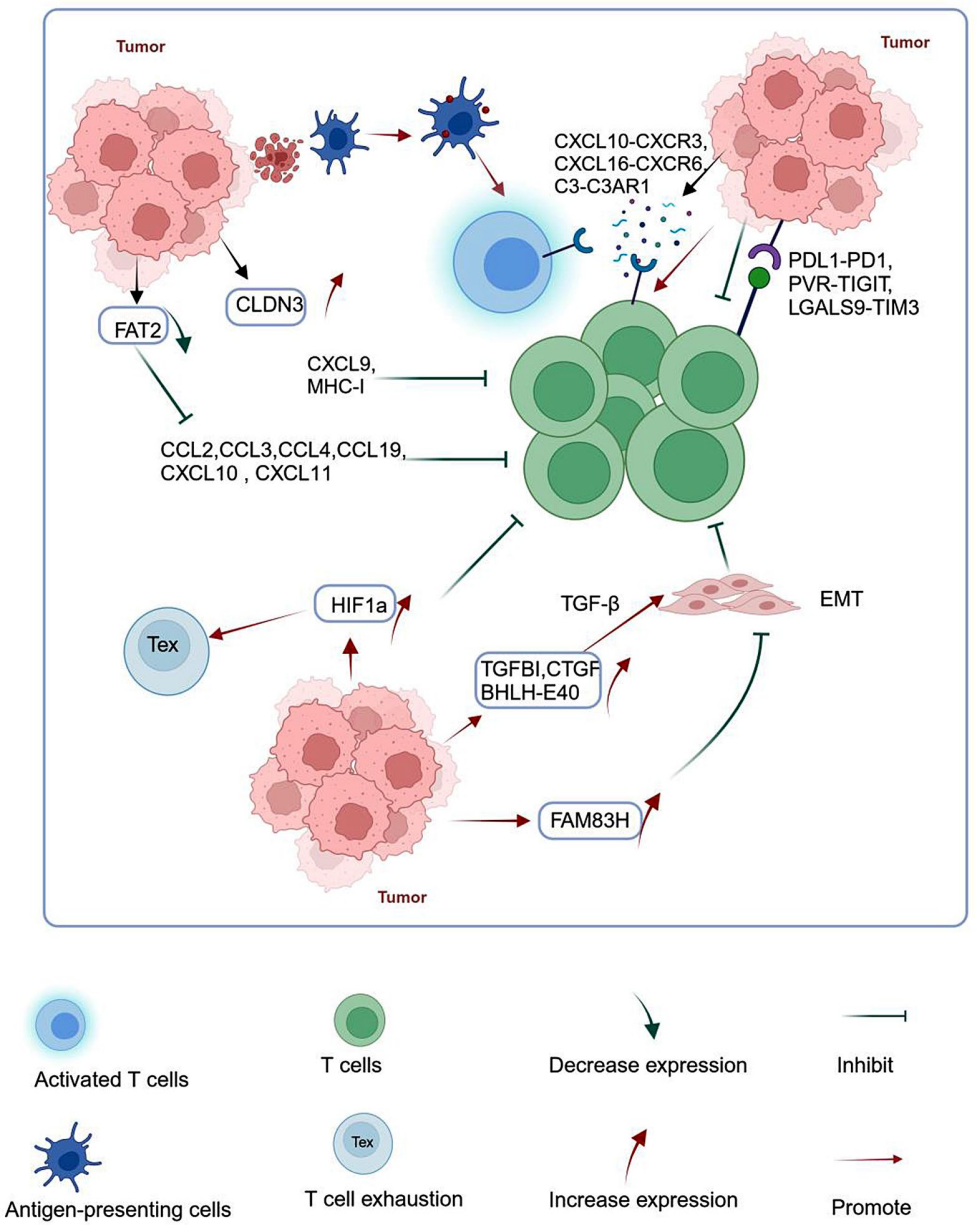
Tumor cells employ multiple mechanisms to evade T-cell killing. (Figure created with BioRender.com). Tumor cells significantly attract T-cell infiltration through interactions, such as CXCL10-CXCR3 and CXCL16-CXCR6, as well as complement factors (e.g., C3-C3AR1). Additionally, they can evade immunity through immunosuppressive interactions with T cells, such as PDL1-PD1, PVR-TIGIT, and LGALS9-TIM3. Tumor cells can express genes responsive to TGF-β, including *TGFBI*, *CTGF*, and *BHLH-E40*, which activate the TGF-β pathway to induce epithelial-mesenchymal transition (EMT), inhibiting T-cell infiltration. Similarly, the *FAM83H* gene can serve a similar role. Tumor cells may inhibit CD8 + T-cell recruitment by reducing *FAT2* gene expression, as well as negatively regulating chemokines such as CCL2, CCL3, CCL4, CCL19, CXCL10, and CXCL11 through various mechanisms. Additionally, they may overexpress *CLDN3*, which inhibits the expression of MHC-I and CXCL9, reducing CD8^+^ T-cell infiltration in tumor tissue. Furthermore, tumor cells can decrease T-cell infiltration and enhance T-cell depletion by activating *HIF1a*

The formation of “hot” and “cold” tumors can be analyzed by examining the interactions between T lymphocytes and other cells within the TME. ScRNA-seq technology holds a distinct advantage in this context. Here, we initially focused on the immune interactions that occur between other tumor cells and T lymphocytes (Fig. 2).

The activation and recruitment of lymphocytes by tumor cells play a critical role in the development of “hot” tumors. The primary catalyst for lymphocyte infiltration is the expression of antigens on the surfaces of tumor cells [55]. Additionally, certain cytokines can be expressed to facilitate the recruitment of T cells. Jin et al. [47] revealed that tumor cells can attract T cells to infiltrate the tumor through diverse interactions, including CXCL10–CXCR3, CXCL16–CXCR6, and C3–C3AR1 (complement factors), in nasopharyngeal carcinoma tissues [14]. Chen et al. [18] identified a specific population of tumor cells with a high expression of chemokines, including CCL20, CCL19, and CXCL10, within nasopharyngeal carcinoma tissues. These chemokines can further enhance the infiltration of T cells. Tumor cells with high levels of *FAT2* expression also show a significant upregulation of the chemokine genes, including CCL2, CCL3, CCL4, CCL19, CXCL10, and CXCL11, in lung adenocarcinoma tissues [56].

The use of high-resolution scRNA-seq enables the classification of tumor cells into distinct subtypes to facilitate the understanding of the effect of various tumor cells on T-cell infiltration. Hara et al. [18] discovered a mesenchymal-like state of tumor cells in gliomas, which correlated with increased cytotoxicity of T cells. Baldominos et al. [57] identified a specific tumor cell phenotype known as the “intratumorally quiescent phenotype” in primary TNBC. This phenotype shows decreased T-cell infiltration and promotes T-cell exhaustion by activating *HIF-1α*.

Tumor cells can influence the infiltration of T cells by selectively increasing the expression of specific genes. Jerby-Arnon et al. [58] identified a collection of gene programs responsible for inducing and inhibiting T-cell exclusion in malignant melanoma cells. These gene programs include *p53*, *Myc*, and *DLL3*, which are associated with T-cell exclusion induction, and *HLA-A*, *c-Jun*, *SQSTM*1, and *LAMP2*, which are associated with T-cell exclusion inhibition. The authors further assessed T-cell exclusion programs in 472 tumors and found that “cold” tumors with low T-cell infiltration showed T-cell exclusion. “Cold” tumors had notably higher scores for genes associated with T-cell exclusion compared with “hot” tumors characterized by high levels of T-cell infiltration. Multiple scRNA-seq-based investigations have indicated that tumor cells can modulate T-cell infiltration by selectively upregulating specific genes. Tumor epithelial cells can express genes that respond to TGF-β, including *TGFBI*, *CTGF*, and *BHLH-E40*. Consequently, the TGF-β pathway is activated [59] and the process of epithelial–mesenchymal transition (EMT) is initiated. EMT can inhibit the infiltration of T cells by excluding the antitumor immune cells and upregulating immunosuppressive cytokines. Notably, these mechanisms may play a role in the development of “exclusion tumors.” A negative correlation exists between the abundance of infiltrating T cells and the expression of genes related to EMT in certain cancers, such as colon cancer [60] and NSCLC [61]. Furthermore, the upregulation of the *FAM83H* gene in pancreatic cancer tissues induced EMT and impaired the infiltration of T cells and their antitumor efficacy (particularly CD8^+^ T cells), which was correlated with unfavorable prognostic outcomes [62]. The presence of tumor cells with high levels of *PLCG2* significantly correlates with T-cell dysfunction in small cell lung cancer [63]. Myeloma cells can inhibit T cells by upregulating *FAM3C*, a protein that interacts with inhibitory receptors such as *KIR2DL3* and *CD244* [64].

Lessi et al. [56] found a progressive decrease in the *FAT2* gene expression in tumor epithelial cells with tumor progression in microinvasive BC. Mutations in *FAT1/2/3/4* are correlated with increased T-cell infiltration compared with the wild-type *FAT2*. *FAT2* mutations independently predict good prognoses for patients with lung adenocarcinoma [65]. Tumor cells overexpressing *CLDN3* can inhibit the expression of *CXCL9* and *MHC-I* to reduce the infiltration of CD8^+^ T cells in gastric cancer [66]. The genes with altered expression in tumor cells can be potential prognostic biomarkers to predict the response of patients to immunotherapy. Therefore, specific inhibitors that can transform “cold” into “hot” tumors can be designed, facilitating the use of immunotherapy in a large number of patients.

Tumor cells can also evade immunity through immunosuppressive interactions with T cells, such as PVR–TIGIT, PDL1–PD1, and LGALS9–TIM3 [67, 68]. LGALS9–TIM3 interactions are also associated with Treg expansion and CTL apoptosis after Epstein–Barr virus infection [47]. This may promote the formation of “cold” tumors. Melanoma cells activate the immunoglobulin and ITIM domain (*TIGIT*) by expressing *CD112* and *CD115*, directly suppressing T-cell activity [50].

### Complex interactions between CAFs and T cells

**Fig. 3:**
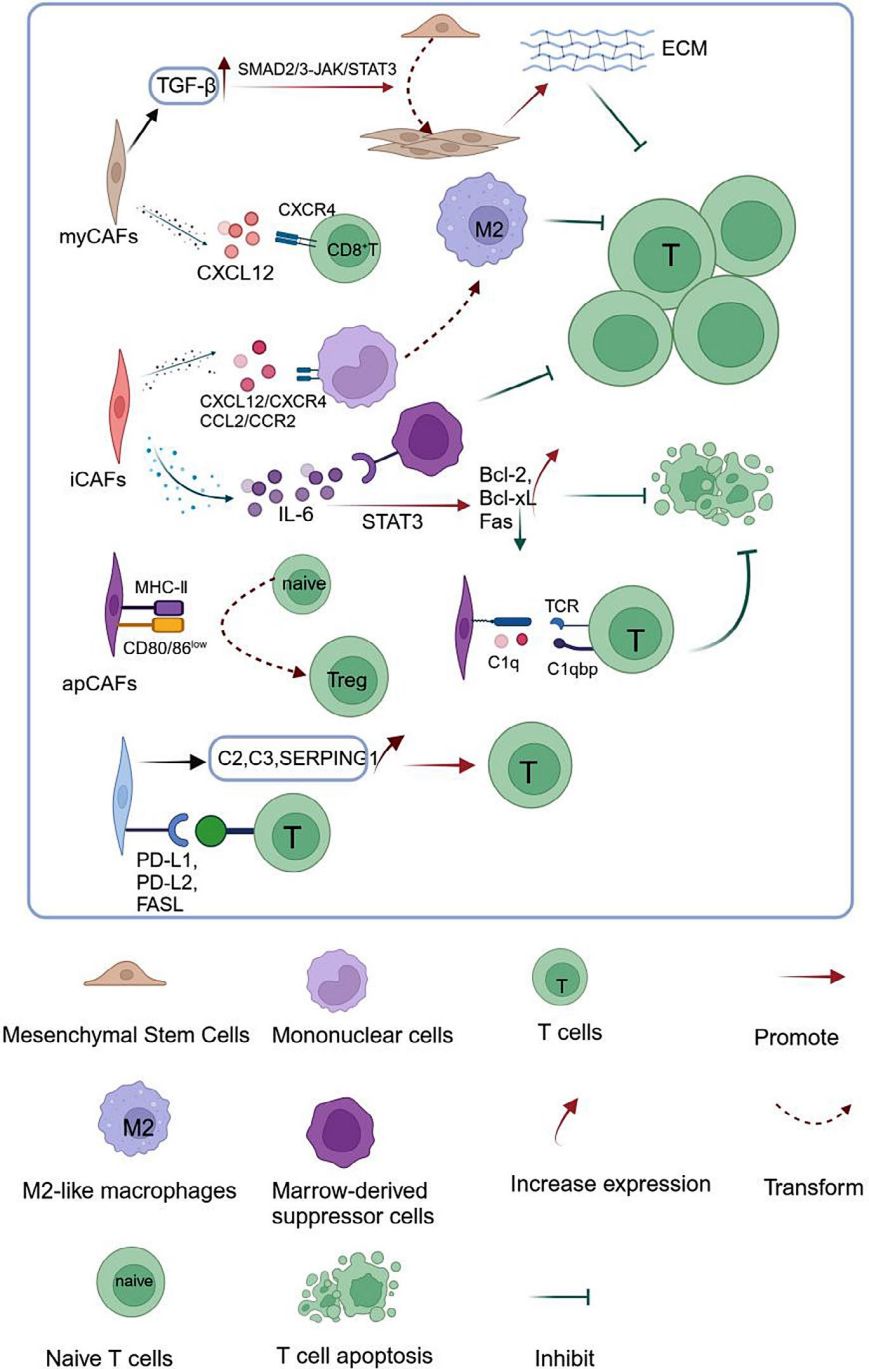
Complex interactions between cancer-associated fibroblasts (CAFs) and T cells. (Figure created with BioRender.com). Myofibroblast CAFs (myCAFs) activate the SMAD2/3-JAK/STAT3 signaling cascade by upregulating TGF-β expression, inducing mesenchymal stem cell differentiation into CAFs and promoting dense extracellular matrix production by CAFs, ultimately blocking T-cell infiltration. They also upregulate CXCL12 expression, recruiting CXCR4^+^ cytotoxic T lymphocytes to the mesenchymal region, preventing T cells from contacting and eliminating cancer cells. Inflammatory CAFs (iCAFs) promote monocyte recruitment and induce their differentiation into M2 macrophages through the CXCL12/CXCR4 or CCL2/CCL2R axis, inhibiting T-cell activation and proliferation. They also recruit myeloma-derived immunosuppressive cells by expressing IL-6 or promote T-cell proliferation in response to T-cell receptor (TCR) stimulation. iCAFs prevent T-cell apoptosis through *STAT3*-dependent upregulation of anti-apoptotic factors (such as Bcl-2 and Bcl-XL) and regulation of surface expression of Fas receptors. Antigen-presenting CAFs (apCAFs) can induce the conversion of naïve CD4^+^ T cells into regulatory T cells (Tregs) in an antigen-specific manner. They can also cause T-cell incompetence or induce Treg formation by lowering the expression of co-stimulatory molecules (e.g., CD40, CD80, and CD86). apCAFs can directly activate the TCR of effector CD4^+^ T cells and promote T-cell infiltration through the complement pathway (C2, C3, and SERPING1). Furthermore, they can directly inhibit the cytotoxic effects of tumor antigen-specific CD8^+^ T cells by expressing ligands such as PD-L1, PD-L2, and FASL

The interactions between stromal and T cells are crucial to the development of tumor immune phenotypes. Stromal cells can regulate the activity of T cells, resulting in different T-cell infiltration patterns [44]. The most predominant stromal cells in the majority of solid tumors are CAFs [69]. Several authors have suggested a significant heterogeneity in CAFs present in the TME using scRNA-seq and suggested three major CAF subtypes [70–72]: cancer-associated myofibroblasts (myCAFs; *αSMA*^high^/*IL-6*^low^), inflammatory CAFs (iCAFs; *αSMA*^low^/*IL-6*^high^), and antigen-presenting CAFs (apCAFs; expressing genes belonging to the MHC-II family). myCAFs are significantly correlated with smooth muscle contraction, focal adhesions, ECM organization, and collagen formation [73]. iCAFs are primarily associated with IFN-γ response and inflammatory pathways, such as IL-2/STAT5, TNF/NF-κB, IL-6/JAK/STAT3, and complement pathways, whereas apCAFs are significantly associated with antigen presentation and processing [52]. The formation of specific immunophenotypes of the tumor significantly correlates with the CAF subtypes. Hornburg et al. [44] found significant differences in fibroblast subsets and T-cell composition between immune-inflamed and immune-excluded tumors in ovarian cancer. Additionally, they observed significant disparities in the spatial distribution of various CAF subtypes within tumor tissues [74], suggesting that different subsets of fibroblasts may influence T-cell infiltration through different possible mechanisms (Fig. 3).

#### myCAFs inhibit T-cell infiltration by forming a physical barrier

Desbois et al. found that exclusionary tumors may target enriched CAFs [75]. Moreover, Mariathasan et al. suggested that exclusionary tumors are significantly associated with the characteristic TGF-β signaling pathway in CAFs [76], and TGFB1 is significantly activated in myCAFs [52]. Cells such as fibroblasts, epithelial cells, and immune cells secrete TGF-β ligands, and TGF-β activates the SMAD2/3-JAK/STAT3 signaling cascade and induces the differentiation of MSCs into CAFs [77]. TGF-β generates a dense ECM by promoting the expansion and activation of CAFs [75]. Immunohistochemical staining revealed that CAFs are predominantly found in the distal stromal region of the tumor [74]. Therefore, myCAFs can form a solid physical barrier to T-cell infiltration by generating ECM [78]. Antibody blockade treatment with a combination of TGF-β and PD-L1 in an exclusion EMT6 mouse BC model significantly downregulated the expression of genes associated with stromal remodeling in CAFs. Notably, T-cell infiltration was significantly increased, mediating tumor regression [76]. Similarly, treatment with the anti-TGF-β/PD-L1 bispecific antibody YM101 promoted “hot tumor” formation with high T-cell infiltration [79]. Overall, the TGF-β signaling pathway and the formation of ECM can inhibit the penetration of T cells into tumors by forming a physical barrier, thereby promoting the formation of “cold” tumors. The “effective inhibition and disruption of the external tumor barrier” is a key area of research to transform “cold” tumors into “hot” ones, allowing patients with highly fibrotic tumors to benefit from immune checkpoint inhibitor (ICI) therapies.

Furthermore, CAFs are significant producers of CXCL12 [80] and mainly attract CXCR4-expressing CD8^+^ T cells. CAFs can misdirect CTLs into the stromal area outside the tumor through the CXCL12–CXCR4 axis, thereby directing T cells to the mesenchymal region and preventing them from recognizing and eliminating cancer cells. Pharmacologic inhibition of the CXCL12–CXCR4 axis promoted CTL infiltration and reduced tumor volume in a mouse pancreatic cancer model [70]. Therefore, CAFs form a chemical barrier by secreting CXCL12 and preventing T-cells infiltration.

Overall, CAFs have the potential to inhibit the immune response against tumors by creating a physical or chemical obstruction and attracting immunosuppressive cells. This ultimately leads to the development of “cold” tumors that are poorly responsive to ICI treatment.

#### Complex molecular interactions between iCAFs and T cells

Grout et al. [52] identified *αSMA*^low^/*IL-6*^high^ CAFs as iCAFs using scRNA-seq. IL-6 concentrations were positively correlated with the enrichment of MDSCs. Furthermore, MDSCs recruited by IL-6 showed a more potent inhibitory effect on the proliferation of CD8^+^ T cells [81]. Some researchers used antibodies to inhibit IL-6R in mice with squamous cell carcinoma. This intervention effectively reduced the accumulation of MDSC subpopulations and concurrently enhanced the antitumor T-cell responses [82, 83]. However, IL-6 may also positively regulate T cells by promoting T-cell proliferation and inhibiting their apoptosis through *STAT3*-dependent upregulation of anti-apoptotic factors (such as Bcl-2 and Bcl-XL) and the regulation of surface expression of the Fas receptors [81]. Therefore, the positive effects of IL-6 on T cells can be considered while developing anti-IL-6 agents for cancer therapy.

ScRNA-seq-derived cell–cell interaction networks revealed that iCAFs communicate with CD8^+^ T cells, CD4^+^ T cells, macrophages, and Tregs through chemokines, inflammatory cytokines, and immune regulation-related receptor–ligand interactions [52]. Immunohistochemical staining further validated the presence of iCAFs near CD8^+^/PD-1^+^ T cells, indicating potential communication between these two cell types to facilitate lymphocyte recruitment [74]. Further, scRNA-seq data analysis revealed that iCAFs expressed high levels of CXCL12, CCL2, CCL7, CXCL1, IL-6, and IL-33 [84]. CXCL12/CXCR4 facilitates the recruitment of monocytes and induce their differentiation into M2-type macrophages with high PD-1 expression and low MHC-II expression [85] or *STAB1*^+^/*TREM2*^high^ lipid-associated macrophages [86], consequently inhibiting the activation and proliferation of T cells. Zeng et al. [87] reported that the use of AMD3100 (a highly specific CXCR4 antagonist) to inhibit the CXCL12/CXCR4 axis in ovarian cancer decreased intratumoral Treg infiltration, increased effector T-cell infiltration, and promoted the polarization of M2 to M1 macrophages in the tumor. iCAFs with high expression levels of CCL2/CCR2 can express monocyte chemotactic protein-1, which can potentially fulfill a similar function [88].

Overall, a comprehensive analysis of the chemokines and cytokines expressed in CAFs will help identify the strategies to transform “cold” into “hot” tumors, thereby increasing the efficacy of immunotherapy.

#### Variable antigen presentation by apCAFs

ScRNA-seq data analysis from multiple studies reveals a population of CAFs expressing MHC-II-like molecules (apCAFs) that play a role in regulating tumor immunity [73]. Notably, apCAFs can present antigens to T cells [89]. They are derived from mesothelial cells, and this transformation is induced by IL-1 and TGF-β. apCAFs can induce the transformation of naïve CD4^+^ T cells into Tregs in an antigen-specific manner [90]. They can also induce T-cell incompetence or Treg formation by expressing co-stimulatory molecules (e.g., CD40, CD80, and CD86) at low levels. Tregs then participate in the immune escape of tumors [91, 92]. Multiple staining of PC tumor tissues revealed that apCAFs were significantly associated with Tregs in patients with pancreatic cancer [90]. However, the regulating effect of apCAFs on tumor lymphocytes is inconclusive. Kerdidani et al. analyzed lung adenocarcinoma tumors and reported that apCAFs can directly activate the T-cell receptor (TCR) of effector CD4^+^ T cells and at the same time produce C1q, which acted on T cell C1qbp to protect them from apoptosis [93]. Therefore, the effects of apCAFs on tumor lymphocytes need to be further studied.

#### Other CAF subpopulations regulate T-cell infiltration

CAFs can promote antitumor immune responses of T cells through the complement pathway. They express the complement genes (such as *C2*, *C3*, and *SERPING1*) that promote T-cell infiltration [47]. CAFs may facilitate immune evasion by expressing various ligands, including PD-L1, PD-L2, and FASL. These ligands directly inhibit the cytotoxic activity of tumor antigen-specific CD8^+^ T cells, thereby promoting immune escape. PD-L1/2 interacts with PD-1 receptors present on the surface of activated CD8^+^ T cells to induce T-cell incompetence [70]. Lakins et al. [94] blocked the activity of PD-L2 or FASL using antibodies in mouse tumor models and observed a reduction in tumor volume and enhanced infiltration and restoration of the killing capacity of antigen-specific CD8^+^ T cells. Further studies on the interactions of fibroblasts with T cells will help screen the patient population that may respond to ICI treatment and devise strategies to increase the efficacy of this treatment in combination with targeted therapies.

### Complex T-cell chemotaxis of tumor-associated endothelial cells (TECs)

**Fig. 4:**
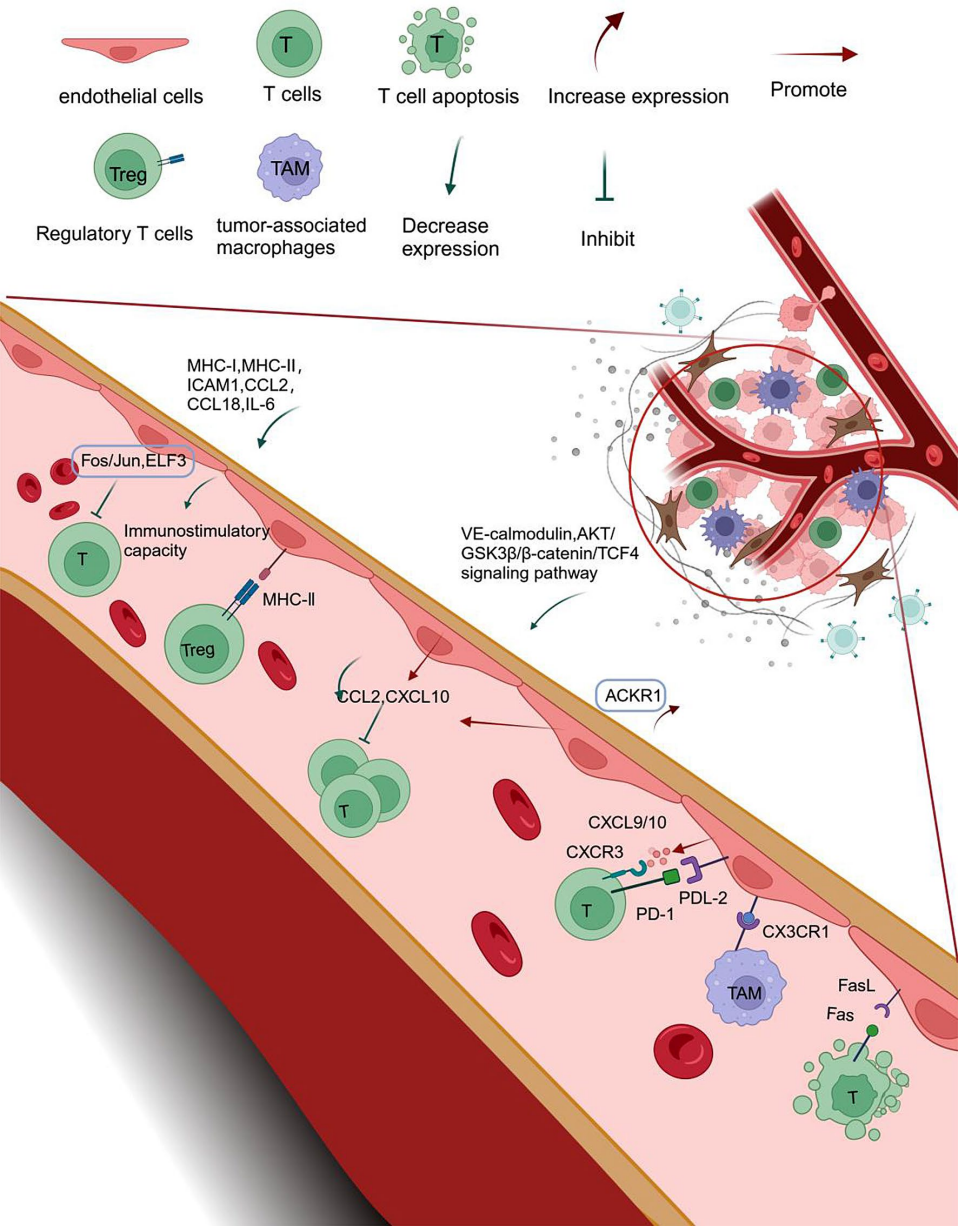
Complex T-cell chemotaxis of tumor-associated endothelial cells. (Figure created with BioRender.com). Tumor-associated endothelial cells (TECs) reduce immunostimulatory capacity by downregulating the expression of genes involved in antigen presentation (MHC class I and II), immune cell homing (ICAM1), and chemotaxis (CCL2, CCL18, IL-6). This downregulation may be associated with the downregulation of *Fos/Jun* and *ELF3*. Additionally, TECs expressing high levels of ACKR1 may inhibit T cells recruitment and infiltration by reducing circulating chemokine concentrations. TECs may also lead to T cell incompetence or induce regulatory T cell formation through high levels of expression of genes for MHC-II-mediated antigen presentation and processing, along with low expression of co-stimulatory molecules such as CD80 and CD86. They can also inhibit T cell activity through PDL2-PD-1 interactions or upregulate the expression of FasL, leading to the killing of T cells through Fas-mediated apoptosis. TECs infiltrating and excluding tumors can recruit CX3CR1 tumor-associated macrophages

TECs (marked by *PECAM1* and *VWF*) [95] are an important component of the TME, promoting angiogenesis and regulating CTLs in the TME. TECs are in direct contact with circulating immune cells in the peripheral blood; therefore, they may not only affect lymphocyte transport but also directly interact with lymphocytes, potentially affecting the formation of “hot” and “cold” tumors (Fig. 4).

TECs may promote “cold tumor” formation by reducing immunostimulatory capacity. Downregulation of MHC-I was associated with “exclusion” and “desert” tumors, rendering CTLs unable to recognize tumor antigens and exert killing effects. ScRNA-seq data analysis revealed the downregulation of gene expression involved in antigen presentation (MHC class I and II), immune cell homing (*ICAM1*), and chemotaxis (*CCL2*, *CCL18*, and *IL-6*) in TECs. The downregulation of *Fos/Jun* and *ELF3* may underlie the reduced immunostimulatory capacity of TECs [96]. Jermaine et al. [97] identified capillary ECs with high expression of genes involved in MHC-II-mediated antigen presentation and processing in lung cancer tissues. However, these cells scarcely express co-stimulatory molecules CD80 and CD86, leading to T-cell incompetence or induce Treg formation. The downregulation of chemotactic genes in TECs may also affect the abundance of CTLs within tumors. CD5-2 is an oligonucleotide drug that specifically increases the expression of VE-calmodulin. Yang et al. [98] conducted an scRNA-seq analysis and observed that CD5-2 administration increased the secretion of chemokines, such as CCL2 and CXCL10, by TECs. These chemokines are involved in leukocyte migration, specifically promoting the infiltration of CD8^+^ T cells. In vitro and in vivo mechanistic studies indicate that the upregulation of CCL2 expression is dependent on the expression of VE-calmodulin and subsequent activation of the AKT/GSK3β/β-catenin/TCF4 signaling pathway. Therefore, the therapeutic targeting of VE-calmodulin is of considerable interest in this context. Hu et al. [99] identified a cluster of ECs expressing high levels of ACKR1 in ccRCC. ACKR1 is a high-affinity nonspecific receptor for inflammatory chemokines. Girard et al. [100] also identified a tumor-associated non-high endothelial venous endothelial cell (TA-EC) that expresses *ACKR1* and *SELP* at high levels and genes related to the blockage of T-cell homing (*EDNRB*) at low levels. The authors suggested that TA-EC can increase the infiltration of stem-like CD8^+^ T cells and decrease the proportion of exhausted CD8^+^ T cells in tumors.

However, TECs also improve immune cell chemotaxis in some “cold” tumors through ascending regulation of the expression of CXCL9–CXCR3 and CXCL10–CXCR3. Therefore, other mechanisms also exist in TECs to impair the immune response against tumors. TECs have an enhanced ability to attract cells and blood vessels compared with non-malignant tissue-derived endothelial cells, but they suppress immune cell activity through PDL2–PD1 interactions [47]. The TME promotes FasL expression on TECs, enabling them to kill CTLs through Fas-mediated apoptosis. FasL expression on endothelial cells is downregulated by the inhibition of *VEGF-A* or cyclic oxygenase [101]. In addition, in exclusion tumors, TECs expressing *CCL21* at high levels can recruit B cells expressing *CCR7*, whereas TECs in infiltrating and exclusion tumors can recruit *CX3CR1*^+^ TAM-like cells [44]. Therefore, the role of TECs in T-cell infiltration is complex, and targeting TECs can be a promising therapeutic strategy for cancer.

### Myeloid immune cells regulate T-cell infiltration

#### Macrophages are a double-edged sword during T-cell infiltration

**Fig. 5:**
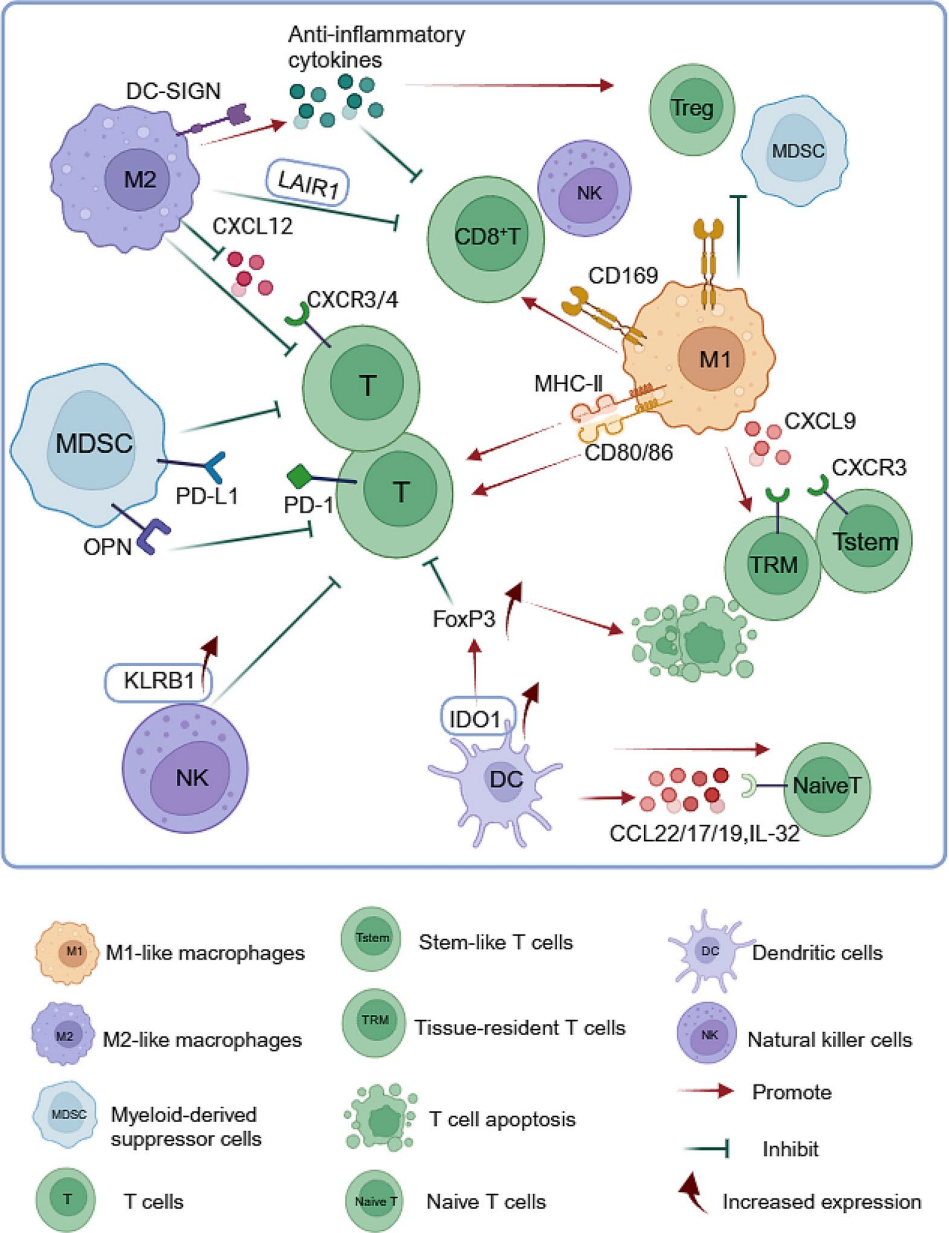
Myeloid immune cells regulate T-cell infiltration. (Figure created with BioRender.com). M1-like tumor-associated macrophages (TAMs) express high levels of *CXCL9,10,11* and *12* and recruit stem cell-like CD8^+^ or CD8A^+^ tissue-resident T cells via the CXCL9-CXCR3 axis. CD169 macrophages promote tumor microenvironment reprogramming by recruiting CD8^+^ T/NK cells and inhibiting the accumulation of myeloid-derived suppressor cells (MDSCs)/regulatory T cells (Tregs). M1-like TAMs also highly express MHC-II, CD68 markers, and CD80 and CD86 co-stimulatory molecules, promoting the recruitment of T cells through local tumor antigen presentation. M2-like TAMs, expressing high levels of DC-SIGN, may promote Treg accumulation and suppress T-cell infiltration by directly engaging in the expression and secretion of multiple anti-inflammatory cytokines, and inhibit T cells activity via the PD-L1/PD-1 pathway. They may also downregulate the CXCL12-CXCR3 and CXCL12-CXCR4 axes to suppress T cells infiltration, upregulate CD86-CTLA4 to inhibit T cells activity, and possibly block CD8^+^ T-cell infiltration via GRN-TNFRSF1A interaction or LAIR1. MDSCs can mediate CD8^+^ T cell incompetence via the PD-L1/PD-1 pathway, and express OPN proteins to interact with PD-1^+^ T cells infiltrating the tumor, promoting tumor immune escape. Dendritic cells can express CCL22, CCL17, CCL19, and IL-32, recruiting naive T cells; they also induce T cell inactivation and apoptosis by increasing *FOXP3* expression through high levels of *IDO1* expression. Natural killer (NK) cells stimulate *BDCA3*^+^ dendritic cells by producing FLT3LG, increasing T-cell infiltration, but they also inhibit the anti-tumor effect of T cells by expressing the *KLRB1* gene (encoding *CD161*)

TAMs are an important component of the TME involved in “hot” and “cold” tumor formation. The microenvironment of solid tumors releases different cytokines (such as CCR2 [102] ) that recruit circulating monocytes and induce them to polarize into antitumor M1-type (marked by *CXCL9*, *CXCL10*, *CCL5*, *STAT1*) or protumor M2-type (marked by *CCL22*, *MMP9*, and *MMP12*) macrophages [103] (Fig. 5).

ScRNA-seq analysis indicated high expression levels of CXCL9, CXCL10, CXCL11, and CXCL12 in M1-type TAMs. These TAMs recruit stem-like CD8^+^ [104] or CD8A^+^ tissue-resident [105] T cells through the CXCL9–CXCR3 axis to enhance patient responses to ICI treatment. Analysis of a large cohort of patients with lung cancer revealed that high levels of M1-type TAMs expressing CXCL9 were associated with strong antitumor immune responses and better prognoses [103]. In addition, macrophages specifically express CD169 in various solid tumors. Hornburg et al. [44] found that *CD169*^*+*^ macrophages can recruit T cells in the same manner in ovarian tumors and such macrophages are mainly enriched in exclusion and infiltrating tumors. *CD169*^*+*^ macrophages promote TME reprogramming by recruiting CD8^+^ T/NK cells and inhibiting the accumulation of MDSCs/Tregs [106]. In addition, M1-type TAMs highly express MHC-II, CD68 markers, and CD80/86 co-stimulatory molecules, and the local presentation of tumor antigens may contribute to the recruitment of T cells [103].

In contrast, M2-type TAMs may enhance the expression of anti-inflammatory cytokines and chemokines associated with tumor invasion and metastasis [107]. Hu et al. identified a type of TAMs expressing high levels of DC-SIGN using scRNA-seq, and *DC-SIGN*^+^ TAMs showed an M2-like anti-inflammatory phenotype. DC-SIGN is a functional receptor directly involved in the expression and secretion of several anti-inflammatory cytokines that may be associated with reduced CTL infiltration and accumulation of Tregs. *DC-SIGN*^+^ TAMs inhibited CTL activity in the TCGA cohort possibly through the PD-L1/PD-1 pathway. The combined blockade of DC-SIGN and PD-1 can produce a more potent effect of promoting CD8^+^ CTL activation and tumor cell clearance compared with the blockade of PD-1 alone [108]. Obradovic et al. [109] identified a class of *C1Q*^+^ TAMs in ccRCC, expressing high levels of *C1Q*, *APOE*, *TREM2*, and *LILRB5*. These macrophages inhibited intratumor T-cell infiltration by upregulating the immune checkpoints PD-1, PD-L1, and LAG-3. In vitro co-culture experiments also demonstrated that *TREM2*^+^ TAMs inhibited T-cell proliferation, and lowering *TREM2* expression reversed these effects [110]. Qi et al. [111] identified a class of *SPP1*^+^ TAMs in colorectal cancer, expressing high levels of *SPP1* and the scavenger receptor *MARCO*. These macrophages promoted the proliferation of extracellular matrix by interacting with *FAP*^+^ CAFs via *TGFB1*, thereby inhibiting the infiltration of T cells within the tumor. Ho et al. [12] found that M2-type TAMs secreted high concentrations of IGF-1 and CCL20 and low concentrations of CXCL9 and CXCL10, which inhibited the recruitment of CD8^+^ T cells and promoted the recruitment of Tregs in hepatocellular carcinoma. In addition, TAMs inhibit CTLs infiltration by downregulating the CXCL12–CXCR3 and CXCL12–CXCR4 axes and suppress CTLs activity by upregulating CD86–CTLA4 [47]. TAMs can also promote T-cell rejection through GRN–TNFRSF1A interaction [105]. Tumor-promoting M2-type macrophages may lead to the formation of exclusion tumors by preventing CD8^+^ T cell infiltration through LAIR1. Co-culture of *LAIR1*-knockdown M2-type macrophages with CD8^+^ T cells led to enhanced T cell activation [112]. Recently, Horn et al. [113] investigated the effects of blocking LAIR1 and TGF-β signaling in mouse models of breast and colon cancers. This intervention led to a remodeling of the tumor collagen matrix, resulting in enhanced infiltration and activation of CD8^+^ T cells and replication of M2-type TAMs. Additionally, Katzenelenbogen et al. isolated a class of *Arg1*^+^*TREM2*^+^ regulatory macrophages from tumors. Knockdown of the *TREM2* gene significantly decreased the number of regulatory macrophages and dysfunctional CD8^+^ T cells and increased NK cells and CTLs, thereby increasing the immune responsiveness of tumors [114]. This finding implies that the TREM2 gene can be a potential therapeutic target, and targeting this gene may help increase the effectiveness of ICI treatment. Overall, the effect of TAMs on T-cell infiltration is complex and needs to be further investigated.

#### Regulation of T-cell infiltration by different subpopulations of MDSCs

MDSCs can inhibit the antitumor activities of T and NK cells (Fig. 5) [115–118]. Loeuillard et al. [102] found a lack of T cells in the central part of human cholangiocarcinoma, whereas T cells coexisted with *CD11b*^+^ MDSCs at the tumor margins, suggesting that MDSCs may prevent the infiltration of T cells into the center of the tumor. An *APOE*^*+*^ granulocyte-like MDSC (G-MDSC) subset with an immunosuppressive gene signature was identified using scRNA-seq. In addition, G-MDSCs mediated CD8^+^ T-cell incompetence through the PD-L1/PD-1 pathway. Combined PD-1 treatment and dual inhibition of TAMs and G-MDSCs promoted infiltration and activation of CD8^+^ T cells and enhanced the antitumor effects [119, 120].

Osteopontin (OPN) is a multifunctional phosphoglycoprotein. This protein is an immune checkpoint, which may compensate for PD-L1 function and promote tumor immune escape by interacting with infiltrating *PD-1*^+^ T cells in tumors [121]. Lu et al. [122] reported an increase in the expression levels of *SPP1* and *CD44* genes, responsible for encoding the OPN proteins, in monocytic MDSCs in pancreatic cancer. The expression of the OPN proteins was mainly regulated by the WDR5–H3K4me3 epigenetic axis, and the inhibition of *WDR5* significantly improved the effect of anti-*PD-1* immunotherapy in inhibiting pancreatic cancer growth in vivo. OPN is involved in the evasion of the immune system by tumors through the regulation of macrophage polarization, macrophage recruitment, and the inhibition of T-cell activation within the TME [123, 124].

#### Influence of other immune cells on T-cell infiltration

DCs also influence the infiltration of tumor lymphocytes (Fig. 5). Sathe et al. [125] reported that the TME in gastric cancer was enriched in a subclass of DCs that expressed chemokines associated with naïve T-cell recruitment, such as CCL22, CCL17, CCL19, and IL-32. In addition, this subclass of DCs highly expressed *IDO1*. The main function of IDO1 is to convert tryptophan into an immunosuppressive catabolic metabolite, kynurenine. Notably, depletion of tryptophan or accumulation of kynurenine or both may induce T-cell inactivation and apoptosis by increasing the *FOXP3* expression [126]. Therefore, DC subclasses expressing high levels of *IDO1* may induce T-cell inactivation and apoptosis.

*BDCA3*^+^ stimulatory DCs positively correlated with peritumoral T cells density in melanoma and responses to ICI treatment in the patients. The abundance of stimulatory DCs correlated with the level of FLT3LG produced by NK cells in the TME [127]. In addition, NK cells can also inhibit the antitumor effects of T cells by expressing the *KLRB1* gene (*CD161*). Blocking the CD161 receptor using antibodies enhances the cytotoxic activity of T cells against glioma cells in vitro and improves their antitumor function in vivo [128]. Therefore, increasing the number and enhancing the activation of NK cells in the TME may promote the efficacy of ICI treatment.

In summary, the formation of different TMEs in “hot” and “cold” tumors is closely related to the stromal cells and their cytokine profiles.

## The implications of immunotherapeutic treatment in tumors

Cancer immunotherapies, including ICI therapy and adoptive cell therapy, have the potential to induce durable responses in multiple solid and hematologic malignancies. Of these, ICI therapies are the most widely used and have emerged as a promising strategy for the treatment of several types of cancer [129, 130]. However, only a small proportion of patients benefit from this therapy [131, 132]. Here, we explored the mechanisms of lymphocyte infiltration in “hot” and “cold” tumors from the perspective of tumor, stromal, and immune cells. We then summarized the genes, cytokines, and chemokines involved in this process and elaborated on their potential as prognostic biomarkers and therapeutic targets [133, 134]. This information can provide the baseline to transform immunologically “cold tumors” into “hot tumors” to overcome immune resistance and enhance the effect of immunotherapy. The reprogramming of CAFs through the use of a TGF-β inhibitor, a CXCR4 antagonist, or other similar approaches results in increased infiltration of T-cells, leading to the transformation of “cold tumors” into “hot tumors” and ultimately improving the prognosis. Notably, the administration of the anti-TGF-β/PD-L1 bispecific antibody YM101 [79, 135, 136] and the highly specific CXCR4 antagonist AMD3100 [87] has been shown to significantly contribute to a robust antitumor immune response. Similar to antiangiogenic strategies, enhancing the immunostimulatory capacity of TECs can also lead to an increase in T-cell infiltration, thereby improving the prognosis. VEGF-inhibiting therapy has been shown to decrease the expression of FasL on TECs, while simultaneously promoting T-cell infiltration and activation [101]. Additionally, the administration of the oligonucleotide drug CD5-2 has been found to enhance the secretion of chemokines by TECs, thereby facilitating the infiltration of CD8^+^ T cells [98]. Moreover, altering the landscape of TAMs by using a dual inhibitor that targets DC-SIGN and PD-1 [108], or an anti-TREM2 monoclonal antibody [137], promotes the augmentation and stimulation of T cells. This, in turn, enhances the effectiveness of ICI therapy. To summarize, the comprehensive characterization of the TME using scRNA-seq technology facilitates the identification of novel therapeutic targets, thereby expanding the potential recipients of immunotherapy among patients.

## Prospect

ScRNA-seq can be used to analyze the heterogeneity of immune infiltration within the TME at the single-cell level. This technique has significantly contributed to our comprehension of immune infiltration patterns in the microenvironment of various types of tumors. Moreover, the scRNA-seq data has provided a novel perspective on the interconversion between “hot” and “cold” tumors and tumor immunotherapy. Therefore, scRNA-seq has emerged as a valuable tool in the field of tumor research, extending our understanding of the TME and its implications for cancer treatment. In the future, scRNA-seq technology can be combined with traditional methods to comprehensively study the causes of different immune infiltration patterns in the TME based on existing theories to develop new strategies for improving the therapeutic efficacy of immunotherapy and novel methods for cancer prevention, diagnosis, and treatment.

## Data Availability

The materials that support the conclusion of this review have been included within the article.

## Abbreviations

apCAFs: antigen-presenting CAFs
BC: breast cancer
CAFs: cancer-associated fibroblasts
ccRCC: clear cell renal cell carcinoma
CTL: cytotoxic T cell
DCs: dendritic cells
EMT: epithelial-mesenchymal transition
G-MDSC: granulocyte-like MDSC
ICI: immune checkpoint inhibitor
iCAFs: inflammatory CAFs
LMM: leptomeningeal melanoma metastases
MDSCs: myeloid-derived suppressor cells
MBM: melanoma brain metastases
myCAFs: myofibroblasts
NSCLC: non-small cell lung cancer
scRNA-seq: single-cell RNA sequencing
TCGA: The Cancer Genome Atlas dataset
TECs: tumor-associated endothelial cells
TNBC: triple-negative breast cancer
TME: tumor microenvironment
TA-EC: tumor-associated non-high endothelial venous endothelial cell
TAMs: tumor-associated macrophages
TCR: T-cell receptor
Tregs: regulatory T cells
